# Eucain Lacate in Nose and Throat Operations

**Published:** 1906-03

**Authors:** 


### ABSTRACTS.

### Eucain Lacate in Nose and Throat Operations.

T. J. HARRIS, M.D., NEW YORK, (*American Medicine,* December 30, 1905) says that Lemare, in a recently published monograph, enumerates the various forms of intoxication which may occur from the local use of cocain,— i. e. (a) disturbances of the circulation; (b) psychic and sensory symptoms; (c) motor troubles; (d) disturbances of the respiration. In the last two years the possibility of accidents has demonstrated itself to him most forcibly in 5 cases. He has therefore studied in hospital and office, beta-eucain lactate in solutions of different strengths. The fact that it produces no hyperemia, ischemia or shrinkage is of decided advantage in certain cases, as of polypoid growths, in which cocain may cause shrinkage or disappearance of the mass, making its removal impossible. When contraction is required, eucain may be combined with adrenalin. Eucain lactate can be used in 25% solution.

He has used it in some 40 to 50 different cases, including 8 for correction of the nasal septum by the Roe method and 4 cases by the Freer method of submucous resection. Here, a 10-15% aqueous solution was applied on cotton for a space of 10 minutes combined with or followed by 1.5000 adrenalin. Just before operating, 20 to 30 minims of a 1% eucain solution combined with adrenalin (eucain lactate, 8 gr.; sodium chlorid, 21 gr.; boiling water, 14 dr.; adrenalin, 18 min.) were injected into the mucous membrance of the nasal septum. In no cases was much pain complained of, and in most there was none at all.

Once a 25% solution was used for removing extensive tuberculosis infiltration in the larynx by means of the galvano-cautery, a most painful procedure, requiring complete abolition of all sensation; the patient felt no pain. A 20% solution produced complete anesthesia of the larynx for removing a papilloma. No pain attended the removal of adenoids in the adult, and the removal of the middle turbinated.

In no cases did the slightest discomfort or annoyance attend its use, nor was there anything that could suggest a posible toxic effect.

The impression gained by the author in this series of cases is that eucain lactate is an excellent substitute for cocain and may be employed in the strongest solutions without fear of toxic symptoms. It does not seem to lose its anesthetic qualities under the usual periods of office use. He believes its anesthetic power is not quite so great as that of cocain, and in the more painful operations on the nose it is best used in a correspondingly stronger solution. He recommends a 15-20% eucain lactate solution when a 10% cocain solution would be employed.

CONSERVATIVE TREATMENT OF OCULAR INJURIES.

FRANK ALLPORT, M.D., CHICAGO, in a paper read at the meeting of the American Association of Railway Surgeons, Chicago,

October 4-6, 1905, (*Railway Surgical Journal,* Vol. XII, No. 3, p. 104, November 1905) says: I have seen such surprising results from unguentum Credé in dangerously infected eyes, where suppuration has perhaps already been firmly established that I feel I should mention them in this paper. I usually care for these infected and perhaps suppurating ocular traumas in the orthodox manner, that is, by cold packs, antiseptic irrigation, atropin, argyrol, etc. But I direct that when night comes the eye be thoroughly cleansed, that argyrol and perhaps atropin be used and anything else done that seems advisable. A thick plaster of Credé's ointment is then spread upon a piece of gauze which is placed directly over the closed lids and secured in position by a soft bandage. This is allowed to reman all night and is removed in the morning for the resumption of the daily routine treatment I have never regretted using the ointment and have seen many eyes saved where salvation seemed well-nigh impossible. The amount of discharge is much lessened and it is surprising to see, when the ointment is removed, how clear the eye is and how the small amount of secretion has been drained from the eye to the ointment. I have even used this treatment in gonorrheal ophthalmia and have always been pleased with its effects.

PYRENOL, A CHEMICAL COMPOUND OF SALICYLIC ACID, BENZOIC ACID AND THYMOL.

Dr. Fritz Loeb, of Prof. Ewald's Medical Division of the Augusta Hospital at Berlin, (*Berliner klin. Wochenschrift,* October 10, 1904), says the favorable results from pyrenol obtained in our division induced Prof. Ewald to ask me to continue and report on its employment. A white, crystalline powder of aromatic odor and sweetish taste, it is the finally successful attempt to combine salicylic acid, benzoic acid and thymol with a sodium salt.

Its constituents give the clue to its pharmacodynamic effects. Schlesinger, who introduced it, first used it in neuralgia, but soon recognised its efficacy in asthma and pertussis. Shortly thereafter Sternberg found that it promptly stops the asthma attacks of chronic bronchitis and acts well in cardiac asthma. Then followed in rapid succession the works of Frey, Isenburg, Burchard, Gruenfeld, Komor, Manasse, Frieser and others, whose experience furnishes the following indications:

Affections of the respiratory organs, asthma, pertussis, pneumonia, and acute, chronic and putrid bronchitis.

Rheumatic affections and infectious diseases, acute or chronic articular or muscular rheumatism, pleurisy, angina and gout.

Since Schlesinger has thoroughly tested pyrenol as an antifebrile, antirheumatic and antineuralgic agent in 150 cases, we shall consider only its effects in respiratory diseases. Here it acts solvent and expectorant, limits secretion and soothes cough. In acute bronchitis and pneumonia it is antipyretic, but far less diaphoretic than salicylic acid. Helfer and Gruenfeld found that it prevents heart complications and is valuable in catarrhal and croupous pneumonia and simple pleurisy. Gruenfeld also says that it is specific in pertussis; and we certainly found it as effective as any of the so-called specifics.

In bronchial asthma, pleurisy, bronchitis and influensa, our results were very good indeed. It acted surprisingly well in bed-ridden phthisis cases, promoting expectoration so markedly that many patients demanded it when it had been withdrawn. Pyrenol is indicated in phthisis when there is a dry, troublesome cough. It has no depressant effect on blood pressure or pulse. The stomach stands it very well. To children or sensitives it may be given in raspberry syrup, peppermint water, cold milk or similar corrigens.

We conclude that pyrenol is entirely free from noxious by-effects and is to be warmly recommended in all cases of acute febrile and chronic bronchitis, asthma, especially bronchial asthma, influenza, angina, simple pleurisy, croupous pneumonia and acute broncho-pneumonia. Our maximum adult dose was 67½ grains daily. As pyrenol contains about 40% salicylic acid, this equals about 27 grains of the latter, which is far below the limit of toleration determined by Brugsch for salicylic acid. We obtained the full theraputic effect without any renal irritation.

---

Writing on pyrenol in neuralgia, Dr. Emmo Schlesinger (*Therap. Monatshefte,* January, 1903), states that its important advantages over older remedies were demonstrated in the First Medical Clinic of the Charité Hospital, in the Institute for Medical Diagnosis, and in the Physiologico-Bacteriological Institute of Berlin. Picrkowski's experiments on animals and human beings showed that even doses far greater than are given theraputically cause no ill effect.

The author used the drug in 149 cases, including polyarthritis, rheumatica, acute myalgia, chronic articular and muscular rheumatism, gout, angina, acute bronchitis, influenza, pneumonia, pleurisy, pertussis, asthma, phthisis, neuralgia, migraine and general pain. The last three classes are the most trustworthy tests of the remedy, especially as an analgesic. When cephalic and joint pains and violent neuralgias cease within half an hour there can be no doubt of its effect. Almost without exception doses of 15 to 30 grains once or twice daily acted quickly and effectually. The best results were seen in a neuralgia in which pain had for a week been terrible; it vanished after 2 drams pyrenol, taken in two days, and there has been no relapse.

Apyrexia and cessation of pain were attained in acute articular rheumatism in about three days, with 2½ to 3 drams of the drug. Hot compresses to promote diaphoresis were also used when there was a joint effusion; the result was excellent and there was no subsequent relaxation and weakness, as with salicylic acid. Chronic rheumatism, as well as the headache and joint pain of angina, acute bronchitis and influenza were rapidly improved. In pneumonia the drug diminshed the pleuritic pain and the fever. In the pleurisies 30 grains twice daily for ten days always lessened the exudate. In exascerbations of asthmatic conditions it is a most effective antispasmodic. Its physiological and clinical action differs from that of its constituents, benzoic acid and salicylic acids and thymol. Pyrenol lacks their drawbacks and possesses their virtues in an enhanced degree.

